# HPV knowledge, awareness, and vaccine beliefs among women attending gynecological care in Türkiye: implications for cervical cancer prevention

**DOI:** 10.1186/s13027-026-00803-1

**Published:** 2026-09-25

**Authors:** Figen Efe Camili, Sevde Aksu

**Affiliations:** 1 Faculty of Medicine, Department of Obstetrics and Gynecology, Balıkesir University, Balıkesir, Türkiye; 2 Faculty of Health Sciences, Department of Nursing, Balikesir University, Balikesir, Türkiye

**Keywords:** Human papillomavirus (HPV), HPV vaccination, Vaccine beliefs, Health awareness, Health knowledge, Women’s health

## Abstract

**Background:**

Despite the availability of effective HPV vaccines, vaccination rates remain low in many countries, including Türkiye. Cervical cancer prevention continues to rely largely on opportunistic screening. Although HPV knowledge, awareness, and vaccine beliefs are recognized as important determinants of vaccination behavior, how these factors interact among women attending gynecological care has not been fully elucidated.

**Methods:**

This cross-sectional study included 300 women aged 18–49 years attending the obstetrics and gynecology outpatient clinic of a university hospital in the Marmara region of Türkiye for gynecological examination. Data were collected using a sociodemographic questionnaire, the HPV Knowledge Scale (HPV-KS), the Human Papilloma Virus Awareness and Concern Scale (HPV-ACS), and the Carolina HPV Vaccination Attitudes and Beliefs Scale (CHPV-VABS). Group comparisons, correlation analyses, and multivariate analysis of variance were performed to examine associations between participant characteristics and HPV knowledge, awareness and concern, and vaccine beliefs.

**Results:**

Participants showed limited HPV knowledge (11.01 ± 8.77 out of 33), moderate HPV-related awareness and concern (43.57 ± 9.95), and moderate HPV vaccine attitudes and beliefs (37.18 ± 7.42). HPV vaccination uptake was low, with only 12.3% of participants reporting prior vaccination. Higher educational attainment was associated with greater HPV knowledge scores (*p* < .001), and vaccinated participants had significantly higher knowledge scores than unvaccinated participants (*p* < .001). Education level was also significantly associated with the combined outcome of HPV knowledge, awareness and concern, and vaccine beliefs (F(3, 293) = 2.76, *p*=.044, η²*p* = .05). Correlation analyses showed strong positive associations between HPV knowledge and HPV-related awareness and concern (*r* = .60, 95% CI [0.52, 0.67], *p* < .001) and between HPV-related awareness and concern and vaccine beliefs (*r* = .61, 95% CI [0.53, 0.68], *p* < .001). In contrast, HPV knowledge showed a weak inverse correlation with vaccine beliefs (*r* = − .16, 95% CI [− 0.27, − 0.05], *p* = .005).

**Conclusion:**

Women attending gynecological examinations demonstrated limited HPV knowledge and very low HPV vaccination uptake despite moderate levels of HPV-related awareness and concern. These findings suggest that awareness alone may be insufficient to promote vaccination and highlight the importance of improving HPV knowledge through targeted educational interventions. As higher educational attainment was associated with greater HPV knowledge, integrating structured counseling and evidence-based communication into routine gynecological care may help address knowledge gaps and strengthen cervical cancer prevention efforts in Türkiye.

## Introduction

Human papillomavirus (HPV) is one of the most common sexually transmitted infections (STIs) worldwide and remains a major public health concern because of its causal role in cervical cancer and several other anogenital and oropharyngeal cancers. Twelve HPV genotypes are classified as high-risk due to their oncogenic potential in both women and men, with HPV types 16 and 18 accounting for approximately 70–80% of cervical cancer cases. In 2020, cervical cancer was the fourth most frequently diagnosed cancer among women worldwide, with an estimated 604,127 new cases and 341,831 deaths. The global prevalence of HPV infection ranges from 2% to 44% [1]. In Türkiye, epidemiological data remain limited because HPV infection is not a notifiable disease; however, available studies have reported prevalence estimates ranging from 2.1% to 25.7% [2].

Prophylactic vaccination, together with safe sexual practices, plays a central role in the primary prevention of HPV-related cancers. HPV vaccination programs have been implemented in many countries worldwide, with the greatest protection achieved when vaccination is administered before the onset of sexual activity. Global coverage of the first dose of the HPV vaccine among girls increased from 20% in 2022 to 27% in 2023, reflecting continued progress in vaccination efforts [3]. Furthermore, recent data from the Centers for Disease Control and Prevention (CDC) demonstrated a substantial reduction in cervical precancerous lesions among young women following the introduction of HPV vaccination, providing strong real-world evidence of vaccine effectiveness. Currently, the bivalent, quadrivalent, and nonavalent (nine-valent) HPV vaccines, together with organized cervical cancer screening programs, constitute the cornerstone of cervical cancer prevention.

The success of these preventive strategies, however, depends not only on vaccine availability but also on public acceptance and participation. HPV knowledge and awareness, vaccine-related beliefs (including perceived susceptibility, severity, benefits, and barriers), and HPV-related concern are recognized as important psychosocial determinants of vaccination and screening behaviors [4, 5]. The Health Belief Model (HBM) proposes that preventive health behaviors are shaped by individuals’ perceptions of disease susceptibility and severity, as well as their beliefs regarding the benefits of and barriers to preventive actions. Within the context of HPV prevention, knowledge and awareness influence these perceptions, whereas HPV-related concern may either encourage preventive behaviors or, when excessive, contribute to avoidance and vaccine hesitancy. Therefore, evaluating these psychosocial constructs together may provide a more comprehensive understanding of the factors influencing HPV-related preventive behaviors.

Recent studies in Türkiye have shown considerable variability in women’s HPV knowledge and awareness, while vaccine hesitancy remains common. Knowledge levels are generally reported to be low to moderate, and perceived barriers, risk perception, and concerns regarding vaccine safety have been identified as important factors limiting vaccine acceptance [6, 7]. Similarly, a systematic review covering the period from 2019 to 2024 found that although most Turkish women had heard of HPV, accurate knowledge remained limited and willingness to receive HPV vaccination was low [8].

Comparable findings have been reported internationally. Studies conducted in different countries have consistently shown that knowledge gaps persist despite relatively high levels of awareness, whereas concerns about vaccine safety and misinformation disseminated through social media continue to contribute to vaccine hesitancy. Moreover, HPV-related knowledge, attitudes, and vaccination uptake vary across sociodemographic and cultural settings, highlighting the need for culturally tailored educational and communication strategies [9, 10].

Although public awareness of HPV has increased in Türkiye, HPV vaccination has not yet been incorporated into the National Immunization Program, and vaccination coverage remains low. Financial barriers, limited access to vaccination, and persistent misconceptions regarding vaccine safety and necessity continue to influence vaccination decisions. However, previous studies have generally evaluated HPV knowledge, awareness, concern, and vaccine beliefs separately. Evidence examining the relationships among these psychosocial constructs within the same population, particularly among women attending gynecological care, remains limited. Gynecological consultations represent a valuable opportunity to provide HPV education, recommend cervical cancer screening, and promote HPV vaccination. Therefore, guided by the Health Belief Model, this study aimed to evaluate HPV knowledge, awareness and concern, and HPV vaccine beliefs among Turkish women aged 18–49 years attending gynecological examinations and to examine the relationships among these constructs.

## Materials and methods

### Study design and participants

This cross-sectional study was conducted between September and December 2025 at the Obstetrics and Gynecology outpatient clinic of a university hospital in the Marmara region of Türkiye. The study, which investigated the relationships among HPV knowledge, HPV-related awareness and concern, and HPV vaccine beliefs, was reported in accordance with the Strengthening the Reporting of Observational Studies in Epidemiology (STROBE) guidelines for cross-sectional studies.

Participants were consecutively recruited from women aged 18–49 years attending the outpatient clinic for gynecological examination until the required sample size was achieved. Sample size was calculated using G*Power version 3.1 for multivariate analysis of variance (MANOVA), assuming a medium effect size (f²(V) = 0.25), a significance level of 0.05, and 80% statistical power, yielding a minimum sample size of 300 participants.

Eligible participants were women aged 18–49 years attending the outpatient clinic for gynecological examination, who were able to communicate in Turkish and provided written informed consent. Women with a current or previous diagnosis of gynecological malignancy or those under follow-up for active HPV infection were excluded. Women with a diagnosed psychiatric disorder or ongoing psychiatric treatment and those with cognitive impairment were also excluded. During the study period, 335 women were approached and assessed for eligibility. Of these, 17 did not meet the eligibility criteria, leaving 318 eligible women who were invited to participate. Eighteen women declined participation; consequently, 300 women were enrolled in the study and included in the final analysis. The participant response rate, calculated as the number of enrolled women divided by the number of eligible women invited to participate, was 94.3%. All questionnaires included in the final analysis were complete; therefore, no missing data or imputation procedures were required.

### Data collection

Data were collected through face-to-face interviews conducted by trained researchers in a private setting within the outpatient clinic. Before participation, all eligible women were informed about the study objectives and procedures, and written informed consent was obtained. Participants completed a Descriptive Information Form, the HPV Knowledge Scale (HPV-KS), the Human Papilloma Virus Awareness and Concern Scale (HPV-ACS), and the Carolina HPV Vaccination Attitudes and Beliefs Scale (CHPV-VABS). Completion of all study instruments required approximately 15–20 min.

The Descriptive Information Form was developed by the researchers based on the relevant literature and consisted of seven items assessing participants’ sociodemographic and health-related characteristics, including age, educational level, marital status, employment status, perceived income level, the presence of chronic disease, and previous HPV vaccination. Detailed data on previous HPV infection, prior HPV testing, and abnormal cervical screening results were not collected as part of the Descriptive Information Form and therefore were not included in the analyses.

#### HPV knowledge scale (HPV-KS)

HPV knowledge was assessed using the HPV Knowledge Scale (HPV-KS), originally developed by Waller et al. and culturally adapted into Turkish by Demir [11, 12]. The Turkish version comprises 33 items evaluating knowledge of HPV infection, HPV vaccination, and cervical cancer screening. Each correct response is scored as 1, whereas incorrect and “I don’t know” responses receive 0 points, yielding a total score ranging from 0 to 33. Higher scores indicate greater HPV-related knowledge. In the present study, the internal consistency of the scale was high (Cronbach’s α = 0.87).

#### Human papilloma virus awareness and concern scale (HPV-ACS)

HPV-related awareness and concern were measured using the Human Papilloma Virus Awareness and Concern Scale (HPV-ACS), developed and validated in Turkish by Yılmaz Esencan et al. [13]. The scale consists of 19 items rated on a five-point Likert scale and includes three dimensions: health-related concern, concern about social exclusion, and awareness. Total scores range from 0 to 76, with higher scores indicating greater HPV-related awareness and concern. The Cronbach’s alpha coefficient in the present study was 0.83.

#### Carolina HPV Vaccination attitudes and beliefs scale (CHPV-VABS)

HPV vaccine attitudes and beliefs were assessed using the Carolina HPV Vaccination Attitudes and Beliefs Scale (CHPV-VABS), originally developed by McRee et al. and validated for the Turkish population by Sunar and Kahyaoğlu Süt [14, 15]. The Turkish version contains 16 items organized into four domains: perceived harms, perceived barriers, perceived effects, and uncertainty regarding HPV vaccination. Higher scores reflect more positive attitudes and beliefs toward HPV vaccination. In the present study, the scale demonstrated good internal consistency (Cronbach’s α = 0.83).

### Statistical analysis

Data were analyzed using the Statistical Package for the Social Sciences (SPSS) version 25.0. Descriptive statistics (frequency, percentage, mean, standard deviation) were used to summarize the data. Distributional characteristics of continuous variables were assessed using the Shapiro–Wilk test together with inspection of skewness, kurtosis, histograms, and Q–Q plots. The relationships among HPV Knowledge Scale, Human Papilloma Virus Awareness and Concern Scale, and Carolina HPV Vaccination Attitudes and Beliefs Scale scores were examined using Pearson correlation analysis. Independent-samples t-tests were used to compare mean scale scores according to education level and HPV vaccination status. These analyses were intended as unadjusted group comparisons rather than as models for identifying independent predictors of HPV-related outcomes. Multivariable regression models were not performed because several clinically relevant potential confounders, including previous HPV infection or testing, cervical screening history, sexual and reproductive characteristics, parity, history of sexually transmitted infections, healthcare provider recommendation, and family history of cervical cancer, were not collected. Consequently, a fully adjusted regression model could not be specified without substantial risk of residual confounding. The findings from the t-tests were therefore interpreted as unadjusted associations and not as independent or causal effects. Prior to MANOVA, assumptions of multivariate normality, linearity, absence of multicollinearity, and homogeneity of variance–covariance matrices were assessed. As composite variables, HPV Knowledge Scale (HPV-KS), Human Papilloma Virus Awareness and Concern Scale (HPV-ACS), and Carolina HPV Vaccination Attitudes and Beliefs Scale (CHPV-VABS) scores were evaluated using multivariate analysis of variance (MANOVA). A p-value of < 0.05 was considered statistically significant. No formal adjustment for multiple comparisons was applied. Given the exploratory nature of the analyses, p-values were interpreted as nominal, and findings were considered with caution in the context of multiple testing.

### Ethical considerations

Ethical approval was obtained from the Ethics Committee of Balikesir University (date: 08.08.2025; approval number: 2025/288) and from the administration of the hospital where the study was conducted. The study adhered to the principles of the Declaration of Helsinki. All participants voluntarily agreed to participate. They were informed about the study aims and procedures, and written informed consent was obtained. Participant confidentiality and anonymity were strictly maintained throughout the study.

## Results

The mean age of the participants was 34.55 ± 9.65 years. Most participants had completed high school education or above (72.2%), were married (72.9%), and reported a moderate income level (74.8%). The HPV vaccination rate was 12.3%. The mean HPV Knowledge Scale (HPV-KS) score was 11.01 ± 8.77, indicating a relatively low level of HPV knowledge. The mean Human Papilloma Virus Awareness and Concern Scale (HPV-ACS) score was 43.57 ± 9.95, while the mean Carolina HPV Vaccination Attitudes and Beliefs Scale (CHPV-VABS) score was 37.18 ± 7.42, indicating moderate levels of HPV-related awareness and concern and vaccine-related attitudes and beliefs, respectively (Table 1).

**Table 1:** Mean scores of the HPV knowledge scale, HPV awareness and concern scale, and Carolina HPV vaccination attitudes and beliefs scale

Variable	M	SD	*n*	Min	Max	Skewness	Kurtosis	Mdn	SE_M_
**HPV Knowledge Scale – Total Score**	11.01	8.77	300	0.0	33.0	0.55	-0.31	10.5	0.72
**HPV Awareness and Concern Scale – Total Score**	43.57	9.95	300	21.0	73.0	-0.15	-0.27	44.5	0.81
Awareness subscale	12.97	4.08	300	6.0	35.0	1.08	1.75	13.0	0.33
Concern subscale	15.97	4.83	300	8.0	32.0	0.21	-0.13	16.0	0.39
Social Exclusion subscale	14.63	5.13	300	5.0	25.0	0.05	-0.61	15.0	0.42
**Carolina HPV Vaccination Attitudes and Beliefs Scale – Total Score**	37.18	7.42	300	16.0	63.0	-0.27	0.73	37.5	0.61
Harms subscale	12.92	3.94	300	6.0	25.0	0.2	-0.17	13.0	0.32
Barriers subscale	11.77	3.18	300	5.0	20.0	0.14	0.3	12.0	0.26
Effects subscale	5.52	1.5	300	2.0	8.0	-0.04	-0.4	6.0	0.12
Uncertainty subscale	6.97	1.82	300	3.0	12.0	-0.02	-0.35	7.0	0.15

Pearson correlation analysis revealed a strong positive correlation between HPV knowledge and HPV-related awareness and concern (*r* = .60, 95% CI [0.52, 0.67], *p* < .001). HPV-related awareness and concern was also strongly and positively correlated with HPV vaccination attitudes and beliefs (*r* = .61, 95% CI [0.53, 0.68], *p* < .001), whereas HPV knowledge showed a weak negative correlation with HPV vaccination attitudes and beliefs (*r* = − .16, 95% CI [− 0.27, − 0.05], *p* = .005). The total HPV-ACS score was strongly and positively correlated with the Awareness (*r* = .68, 95% CI [0.61, 0.74], *p* < .001), Concern (*r* = .83, 95% CI [0.79, 0.86], *p* < .001), and Social Exclusion (*r* = .62, 95% CI [0.55, 0.69], *p* < .001) subscale scores. Similarly, the total CHPV-VABS score was strongly and positively correlated with the Harms (*r* = .78, 95% CI [0.73, 0.82], *p* < .001), Barriers (*r* = .76, 95% CI [0.71, 0.80], *p* < .001), and Uncertainty (*r* = .66, 95% CI [0.59, 0.72], *p* < .001) subscale scores and moderately and positively correlated with the Effects subscale (*r* = .49, 95% CI [0.40, 0.57], *p* < .001) (Table 2).

**Table 2:** Pearson correlation results among the HPV knowledge scale, HPV awareness and concern scale, and Carolina HPV vaccination attitudes and beliefs scale

Variable	1	2	3	4	5	6	7	8	9	10
1. **HPV Knowledge Scale – Total Score**	-									
2. **HPV Awareness and Concern Scale – Total Score**	.60*	-								
3. **Carolina HPV Vaccination Attitudes and Beliefs Scale – Total Score**	− .16*	0.61*	-							
4. Awareness subscale	− 0.18	**0.68** ^*^	0.10	-						
5. Concern subscale	− 0.05	**0.83** ^*^	− 0.01	**0.56** ^*^	-					
6. Social Exclusion subscale	0.20	**0.62** ^*^	− 0.05	− 0.01	0.22	-				
7. Harms subscale	− 0.15	0.04	**0.78** ^*^	0.18	0.07	− 0.12	-			
8.Barriers subscale	− 0.15	− 0.02	**0.76** ^*^	− 0.01	− 0.02	− 0.02	.32^*^	-		
9. Effects subscale	0.08	− 0.13	**0.49** ^*^	− 0.14	− .27^*^	0.11	0.09	**0.38** ^*^	-	
10. Uncertainty subscale	− 0.14	0.11	**0.66** ^*^	0.17	0.06	0.01	**0.38** ^*^	.37^*^	.30^*^	-

Note. Values are Pearson correlation coefficients (r). *p < .05

**Fig. 1:**
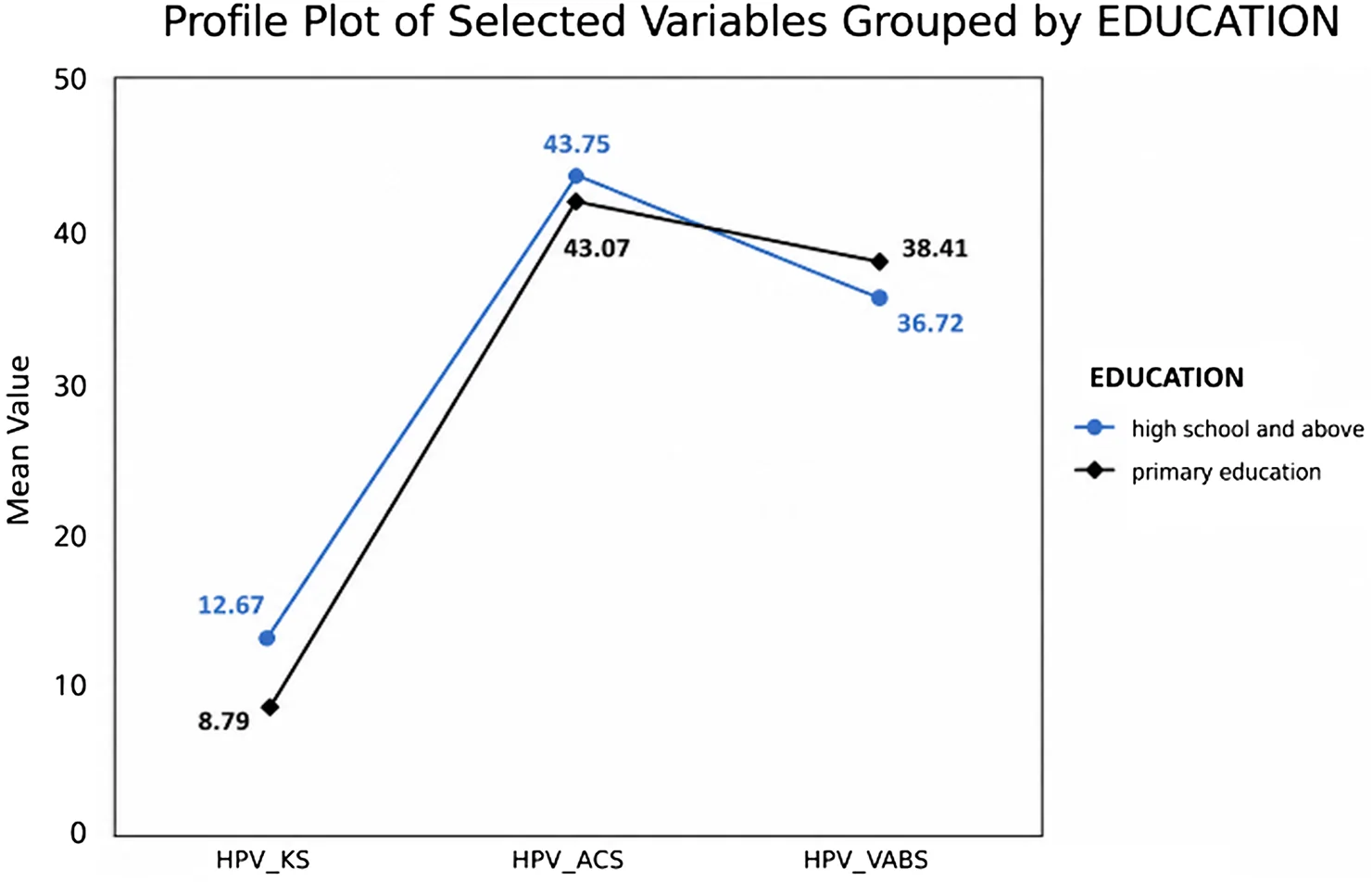
Comparison of HPV-related scale scores according to educational level. Mean scores on the HPV Knowledge Scale (HPV-KS), Human Papilloma Virus Awareness and Concern Scale (HPV-ACS), and Carolina HPV Vaccination Attitudes and Beliefs Scale (CHPV-VABS) are presented for women with primary education and those with high school education or above. Between-group comparisons were performed using independent-samples t-tests. Women with high school education or above had significantly higher HPV-KS scores than women with primary education (12.67 ± 8.20 vs. 8.79 ± 6.61, respectively; p < .001), whereas HPV-ACS and CHPV-VABS scores did not differ significantly between the educational groups. Values are presented as mean ± standard deviation (SD). p< .05 indicates statistical significance

Mean HPV-ACS scores did not differ significantly between women with primary education and those with high school education or above (43.07 ± 10.23 vs. 43.75 ± 9.88, respectively; t = − 0.370, *p* = .711). Similarly, mean CHPV-VABS scores did not differ significantly between the primary education and high school education or above groups (38.41 ± 7.25 vs. 36.72 ± 7.46, respectively; t = 1.137, *p* = .213). In contrast, mean HPV-KS scores differed significantly between the two educational groups (8.79 ± 6.61 vs. 12.67 ± 8.20, respectively; t = − 3.317, *p* < .001), with women who had completed high school education or above demonstrating significantly higher HPV knowledge scores than those with primary education (Fig. 1). However, education level was not associated with HPV-related awareness and concern or HPV vaccination attitudes and beliefs.

**Fig. 2:**
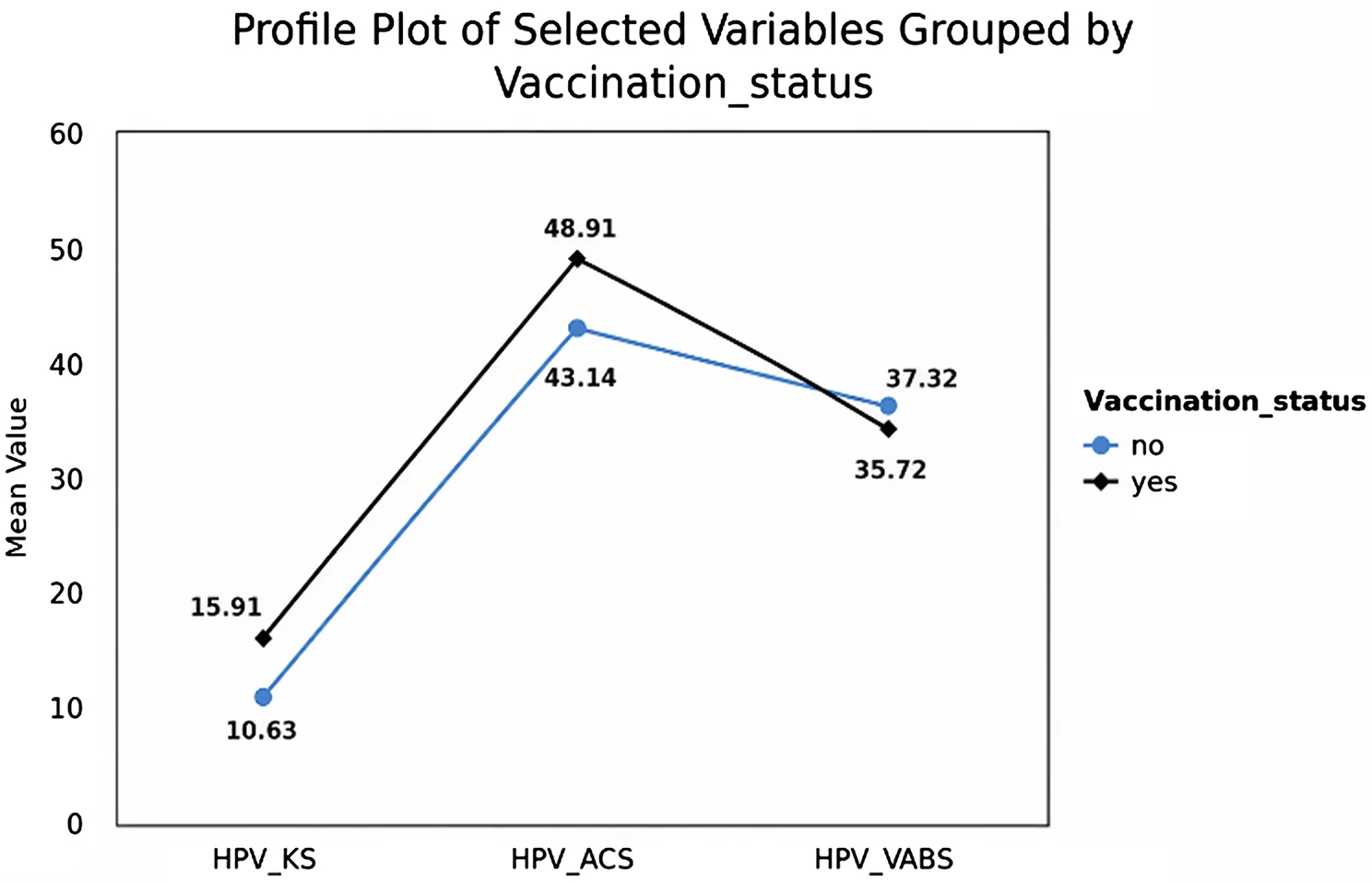
Comparison of HPV-related scale scores according to HPV vaccination status. Mean scores on the HPV Knowledge Scale (HPV-KS), Human Papilloma Virus Awareness and Concern Scale (HPV-ACS), and Carolina HPV Vaccination Attitudes and Beliefs Scale (CHPV-VABS) are presented for HPV-vaccinated and unvaccinated women. Between-group comparisons were performed using independent-samples t-tests. HPV-vaccinated women had significantly higher HPV-KS scores than unvaccinated women (15.91 ± 3.78 vs. 10.63 ± 8.94, respectively; p < .001), whereas HPV-ACS and CHPV-VABS scores did not differ significantly according to vaccination status. Values are presented as mean ± standard deviation (SD). p < .05 indicates statistical significance

The mean of HPV Awareness and Concern Scale (HPV-ACS) was not significantly different between the yes (48.91 ± 9.31) and no (43.14 ± 9.90) categories of vaccination status (t = 1.850, *p*= .064). The mean of Carolina HPV Vaccination Attitudes and Beliefs Scale (CHPV-VABS) was not significantly different between the yes (35.45 ± 5.80) and no (37.31 ± 7.53) categories of vaccination status (t=-0.800, *p*= .420). The mean of HPV Knowledge Scale (HPV-KS) was significantly different between the yes (15.91 ± 3.78) and no (10.63 ± 8.94) categories of vaccination status (t = 3.800, *p* < .001) (Fig. 2). Women who had received the HPV vaccine demonstrated significantly greater HPV knowledge than unvaccinated women. However, vaccination status was not associated with HPV-related awareness and concern or HPV vaccination attitudes and beliefs.

The correlations among the dependent variables were analyzed using Pearson correlation coefficients. The composite variables—HPV Knowledge Scale, HPV Awareness and Concern Scale (HPV-ACS), and the Carolina HPV Vaccination Attitudes and Beliefs Scale (CHPV-VABS)—were evaluated using multivariate analysis of variance (MANOVA). Univariate and multivariate outliers were examined through boxplots and Mahalanobis distance (*p* > .001), and no outliers were detected. Scatterplots indicated an approximately linear relationship among the dependent variables across all categories of the independent variables. Correlations among the dependent variables were below the threshold indicating problematic multicollinearity, supporting the suitability of the data for MANOVA. Box’s M test did not indicate a statistically significant violation of the homogeneity of variance–covariance matrices (*p* > .001). MANOVA results revealed statistically significant differences across education levels in the composite dependent variable (F(3, 293) = 2.76, *p* = .044, Pillai’s Trace = 0.05, η²ₚ = 0.05). The partial eta-squared value (η²ₚ = 0.05) indicated a small-to-moderate multivariate effect associated with education level. Similarly, MANOVA results showed that differences across marital status were also statistically significant for the composite dependent variable (F(3, 293) = 2.64, *p* = .041, Pillai’s Trace = 0.05, η²ₚ = 0.05). The partial eta-squared value (partial η² = 0.05) indicated a small-to-moderate multivariate effect associated with marital status. (Table 3).

**Table 3:** MANOVA results for the HPV knowledge scale, HPV awareness and concern scale, and Carolina HPV vaccination attitudes and beliefs scale

Variable	Pillai’s Trace		F		Hypothesis df		Error df		p		η²ₚ
Education level	0.05		2.76		3		293		.044		0.05
Marital status	0.05		2.64		3		293		.041		0.05
Income level	0.01		0.57		3		293		.634		0.01
HPV vaccination status	0.03		1.50		3		293		.218		0.03

Note. Multivariate effects were evaluated using Pillai's Trace. The dependent variables included the HPV Knowledge Scale (HPV-KS), HPV Awareness and Concern Scale (HPV-ACS), and Carolina HPV Vaccination Attitudes and Beliefs Scale (CHPV-VABS). The reported hypothesis df = 3 corresponds to the approximate F test for the three dependent variables in the SPSS Multivariate Tests output and should not be interpreted as the number of categories of the independent variable

## Discussion

The present study investigated the relationships among HPV knowledge, HPV-related awareness and concern, and HPV vaccine beliefs among women attending gynecological examinations. Three principal findings emerged. First, participants demonstrated limited HPV knowledge despite moderate levels of awareness and concern and moderate HPV vaccine beliefs. Second, HPV vaccination uptake was remarkably low, with only 12.3% of participants reporting previous vaccination. Third, although HPV knowledge, HPV-related awareness and concern, and HPV vaccine beliefs were significantly interrelated, these relationships differed in strength, suggesting that they represent related but distinct psychosocial constructs associated with HPV-related perceptions and preventive behaviors.

Our findings suggest that HPV knowledge, awareness, and vaccine-related attitudes do not necessarily translate into actual vaccination behavior. Although participants demonstrated moderate levels of awareness and generally neutral-to-positive attitudes toward HPV vaccination, vaccine uptake remained remarkably low. This observation is consistent with accumulating evidence showing that awareness can promote preventive health behaviors only when accompanied by accurate knowledge and practical opportunities to access vaccination [17, 18]. Systematic reviews and meta-analyses have similarly demonstrated that greater HPV knowledge is associated with more favorable vaccination attitudes and stronger vaccination intentions, whereas misinformation remains one of the principal determinants of vaccine hesitancy [10, 19–21]. Educational interventions have been shown to improve both HPV-related knowledge and attitudes [22]; however, increasing evidence suggests that these improvements do not consistently translate into higher vaccination uptake [23, 24]. Collectively, these findings indicate that educational interventions alone are unlikely to overcome barriers to HPV vaccination without simultaneously addressing structural and psychosocial determinants of vaccine acceptance.

One of the most noteworthy findings of this study was the differing strength of the relationships among HPV knowledge, HPV-related awareness and concern, and HPV vaccine beliefs. Although HPV knowledge was strongly associated with HPV-related awareness and concern, its relationship with vaccine beliefs was comparatively weak, whereas HPV-related awareness and concern demonstrated a much stronger association with vaccine beliefs. Notably, the association between HPV knowledge and vaccine beliefs was weak and negative (*r* = − .16, 95% CI [− 0.27, − 0.05], *p* = .005), a finding that warrants cautious interpretation. Given the small magnitude of the correlation, this result should not be interpreted as evidence that greater HPV knowledge leads to less favorable vaccine beliefs. One possible explanation may relate to the multidimensional nature and scoring characteristics of the CHPV-VABS, which assesses perceived harms, barriers, effects, and uncertainty rather than factual knowledge alone. Women with greater HPV knowledge may also be more aware of vaccine-related controversies, potential adverse effects, costs, or access barriers, which could contribute to more critical or nuanced responses to vaccine-belief items. Cultural and contextual factors specific to the study population, including concerns regarding vaccine safety, social perceptions of HPV as a sexually transmitted infection, and the out-of-pocket availability of HPV vaccination in Türkiye, may also contribute to this pattern. In addition, characteristics of this single-center clinical sample and unmeasured factors such as previous HPV-related experiences or healthcare provider recommendations may have influenced the observed association. Therefore, the weak inverse correlation should be regarded as exploratory and context-dependent rather than as evidence of an adverse effect of HPV knowledge on vaccine attitudes. Further studies are needed to examine this unexpected relationship using subscale-level analyses and more diverse populations. Taken together, these findings suggest that factual knowledge alone may not correspond directly to more favorable vaccine beliefs and that these constructs may capture different dimensions of HPV-related perceptions. Instead, vaccine beliefs appear to be influenced by a broader set of psychosocial factors, including perceived susceptibility, perceived benefits, perceived barriers, social norms, and confidence in vaccine safety. These findings are consistent with the Health Belief Model, which proposes that knowledge influences preventive behavior indirectly through perceptions of susceptibility, severity, benefits, barriers, and cues to action rather than acting as an independent determinant of vaccination [4, 5]. Consequently, interventions designed to improve HPV prevention should extend beyond information provision and incorporate strategies that address emotional, social, cultural, and structural determinants of vaccination behavior [7, 25, 26].

The low HPV vaccination rate observed in this clinical sample should be interpreted cautiously and within the broader healthcare context in Türkiye. The HPV vaccination rate observed in the present study (12.3%) appears somewhat higher than rates reported in several previous studies conducted among women in Türkiye. Recent clinic-based studies have reported vaccination rates of 7.0% and 9.7%, while a regional study among women aged 15–49 years reported a rate of 3.2% [6, 27]. However, these estimates were derived from different study populations, geographical regions, and sampling approaches and should not be interpreted as national prevalence estimates. Importantly, HPV vaccination has not been incorporated into the routine National Immunization Program in Türkiye, and directly comparable nationally representative vaccination coverage data for adult women remain limited. Therefore, the 12.3% vaccination rate observed in our single-center tertiary-care sample should not be considered representative of HPV vaccination coverage among women in Türkiye. Women attending a university gynecology clinic may differ from the general female population in healthcare-seeking behavior, access to health information and preventive services, socioeconomic characteristics, and opportunities to receive counseling regarding HPV vaccination. Population-based studies using nationally representative sampling are needed to establish reliable HPV vaccination coverage estimates among women in Türkiye. Although participants demonstrated moderate awareness and generally favorable vaccine attitudes, vaccination uptake remained limited, indicating that structural barriers may outweigh individual knowledge and beliefs. HPV vaccination has not yet been incorporated into the Turkish National Immunization Program, requiring individuals to obtain the vaccine through out-of-pocket payment. Previous studies have identified financial constraints, limited accessibility, inconsistent healthcare provider recommendations, and misconceptions regarding HPV vaccination as potential barriers to vaccine uptake. However, these factors were not directly assessed in the present study and therefore cannot be inferred as explanations for the low vaccination rate observed in our sample. Similar barriers have been reported in other middle-income countries where HPV vaccination depends primarily on individual access rather than publicly funded immunization programs. In contrast, countries that have integrated HPV vaccination into their routine national immunization schedules have achieved substantially higher vaccine coverage and have subsequently reported marked reductions in HPV infection, cervical precancerous lesions, and cervical cancer incidence. This finding is particularly concerning given the World Health Organization’s global strategy to eliminate cervical cancer, which aims to achieve 90% HPV vaccination coverage among girls by 15 years of age. The low vaccination uptake observed in the present clinical sample highlights the need for further investigation of individual, healthcare-related, and structural barriers to HPV vaccination in Türkiye. Although national vaccination policy was beyond the scope of the present study, the low vaccination uptake observed in this clinical sample highlights the importance of addressing potentially modifiable barriers to HPV prevention. Within clinical practice, strengthening evidence-based counseling, addressing misconceptions regarding HPV vaccination, and providing individualized vaccine recommendations may represent feasible strategies to support informed vaccination decisions. Broader policy-level strategies, including vaccine financing and integration into national immunization programs, should be evaluated through population-based, health policy, and cost-effectiveness studies specifically designed to address these questions.

In the present study, women with higher educational attainment demonstrated significantly greater HPV knowledge, and education level was also associated with the combined HPV-related outcome in the multivariate analysis. However, these findings should be interpreted cautiously because several potentially relevant clinical, reproductive, behavioral, and healthcare-related confounders were not measured. Therefore, the observed association between education and HPV-related outcomes should not be interpreted as evidence of an independent or causal effect. These findings are consistent with previous research reporting associations between higher educational attainment and greater health literacy, more accurate risk perception, and more favorable HPV-related perceptions [8, 28, 29]. Although the observed effect size was modest, the association between educational attainment and HPV knowledge may have public health relevance by identifying groups that could particularly benefit from accessible and tailored HPV education. Given the cross-sectional design, causal relationships cannot be established; nevertheless, these findings emphasize the importance of developing accessible and comprehensible educational interventions that reach women across all educational backgrounds.

Marital status was also associated with the composite HPV-related outcome, although the observed effect size was small and should be interpreted cautiously. Given the possibility of residual confounding, this association should not be interpreted as an independent effect. Marital status may instead reflect broader cultural, socioeconomic, sexual, and reproductive contexts associated with women’s perceptions of HPV and preventive healthcare [16, 30, 31]. Future studies should evaluate marital status together with sexual behavior, parity, reproductive intentions, previous HPV infection and testing, and healthcare provider recommendations to better understand these relationships.

From a clinical perspective, gynecological outpatient clinics provide an important setting for HPV education because they offer direct contact with women eligible for cervical cancer prevention services. Routine consultations may provide opportunities to assess HPV knowledge, address misconceptions regarding vaccine safety and effectiveness, and provide individualized evidence-based counseling regarding HPV vaccination. Given the cross-sectional and single-center design of the present study, broader conclusions regarding national vaccination policy cannot be drawn from these data. Future population-based studies should investigate structural barriers to HPV vaccination, including affordability and accessibility, while health policy and cost-effectiveness studies are needed to evaluate potential population-level vaccination strategies in Türkiye.

This study has several strengths. It simultaneously evaluated multiple HPV-related psychosocial constructs using validated and reliable measurement instruments within a clinical population of women attending gynecological outpatient services. Recruiting women during routine gynecological visits enhanced the clinical relevance of the findings because these consultations provide important opportunities for HPV education, cervical cancer screening, and vaccination counseling.

Several limitations should be considered when interpreting the findings. First, the cross-sectional design precludes causal inference regarding the observed associations. Second, participants were consecutively recruited from a single tertiary university hospital in the Marmara region of Türkiye, which may have introduced selection bias and limited the external validity of the findings. Women attending a tertiary gynecological outpatient clinic may differ systematically from women in the general population in terms of healthcare-seeking behavior, access to health services, health literacy, socioeconomic characteristics, and previous exposure to HPV- or cervical cancer-related information. Therefore, the observed levels of HPV knowledge, awareness and concern, vaccine beliefs, and vaccination uptake may not be representative of women receiving care in primary or secondary healthcare settings, those with limited access to healthcare, or women living in other geographical and sociocultural regions of Türkiye. Although consecutive sampling may reduce investigator-driven participant selection within the study setting, it does not eliminate selection bias arising from the single-center, healthcare-seeking study population. Accordingly, the findings should be interpreted as reflecting women attending gynecological care at the study center rather than the broader population of women in Türkiye. Future multicenter studies using population-based or probability sampling across different healthcare settings and geographical regions are needed to improve representativeness and external validity.

Third, several potentially important clinical, reproductive, and healthcare-related variables that may confound the associations between sociodemographic characteristics and HPV-related outcomes were not collected in this study. These include previous cervical cancer screening and Pap smear history, abnormal cervical screening results, prior HPV testing or HPV infection, sexual and reproductive history, parity, history of sexually transmitted infections, healthcare provider recommendation regarding HPV vaccination, and family history of cervical cancer. These factors may influence HPV knowledge, awareness, vaccine beliefs, and vaccination behavior independently of the sociodemographic characteristics examined. Consequently, residual confounding cannot be excluded, and the observed associations, particularly those involving education and marital status, should be interpreted cautiously rather than as independent or causal effects. Future studies should incorporate these clinically relevant variables and use multivariable analytical approaches to better identify factors independently associated with HPV-related knowledge, perceptions, and vaccination behavior.

Fourth, no formal adjustment for multiple comparisons was applied. Given the number of statistical tests performed, the possibility of an increased type I error cannot be excluded; therefore, statistically significant findings should be interpreted cautiously and considered exploratory where appropriate. In particular, the weak inverse correlation between HPV knowledge and vaccine beliefs (*r* = − .16) should be regarded as an exploratory association rather than a strong or clinically established relationship. Finally, all data were self-reported and may therefore have been influenced by recall and social desirability bias.

## Conclusion

This study demonstrated that women attending gynecological examinations at a tertiary university hospital in Türkiye had limited HPV knowledge despite moderate levels of HPV-related awareness and concern, and HPV vaccination uptake remained remarkably low. Although HPV knowledge, HPV-related awareness and concern, and vaccine beliefs were significantly interrelated, they appeared to represent distinct but interrelated psychosocial constructs associated with HPV-related perceptions and preventive behaviors. These findings highlight a substantial gap between HPV awareness and actual vaccination behavior, suggesting that improving knowledge alone may be insufficient to achieve meaningful increases in vaccine uptake. Within clinical practice, evidence-based education, individualized counseling, and communication approaches addressing vaccine-related concerns and perceived barriers may support informed vaccination decisions. Further population-based research is needed to identify structural and healthcare-related barriers to HPV vaccination and to determine which strategies may most effectively improve vaccine uptake in Türkiye.

## Data Availability

The datasets analyzed during the current study are available from the corresponding author on reasonable request.
